# Gasdermin D mutation protects against renal ischemia reperfusion injury

**DOI:** 10.14814/phy2.70254

**Published:** 2025-04-23

**Authors:** Jennifer S. Y. Li, Aadhar Moudgil, Daniel N. Meijles, Karli Shaw, Sohel M. Julovi, Katie Trinh, Stephen I. Alexander, Natasha M. Rogers

**Affiliations:** ^1^ Centre for Transplant and Renal Research Westmead Institute for Medical Research Westmead Australia; ^2^ Sydney Medical School, Faculty of Health and Medicine University of Sydney Camperdown Australia; ^3^ Department of Renal Medicine Westmead Hospital Westmead Australia; ^4^ Molecular and Clinical Science Institute St George's, University of London London UK; ^5^ Centre for Kidney Research Children's Hospital at Westmead Westmead Australia

**Keywords:** acute kidney injury, gasdermin D, renal tubular epithelial cells

## Abstract

Pyroptosis, the most inflammatory form of cell death, is dependent on membrane pore formation governed by the assembly of cleaved Gasdermin D (GSDMD). We hypothesized that regulated necrosis pathways are crucial in the pathophysiology of acute kidney injury (AKI). Mice with an isoleucine‐to‐asparagine loss‐of‐function mutation in the Gasdermin D gene (GSDMD^I105N/I105N^) generated by ethylnitrosourea‐mutagenesis were subjected to bilateral renal ischemia–reperfusion injury (IRI) with bio‐molecular readouts performed at 24 h. IRI was also performed in mice pretreated with disulfiram. Whole‐body irradiation followed by syngeneic bone marrow transplantation generated chimeric mice prior to IRI. Mice homozygous for the GSDMD I105N mutation were protected from IRI, demonstrating lower serum creatinine and reduced histological injury, as well as decreased pro‐inflammatory cytokine expression and oxidative stress. Chimeric mice showed that this protection was predominantly governed by mutations in the parenchymal tissue, with a potential contribution from the hematopoietic compartment. Pharmacological inhibition of GSDMD pore formation using disulfiram protected against IRI. Manipulation of GSDMD is an attractive target to mitigate inflammation and cellular death following AKI.

## 1 INTRODUCTION

Acute kidney injury (AKI) remains a widespread problem among acutely hospitalized patients, and an inevitable consequence of kidney transplantation (Bagshaw et al., 2007). Recognition of AKI is often poor, with treatment limited to supportive care and renal replacement therapy in severe cases until recovery. Renal tubular epithelial cells (RTEC) are acutely susceptible to injury in AKI because of their high metabolic rate, and both the manner and extent to which these cells die correlate with the degree of inflammatory infiltrate and subsequent tissue injury. Pyroptosis is a highly inflammatory form of cell death; targeting this regulated pathway may provide new treatment targets for AKI. Pyroptosis was first studied in the context of cellular control of microbial infection but is increasingly recognized as important in the basic biology of disease, including kidney disease (Hutton et al., 2016; Krautwald & Linkermann, 2014; Linkermann et al., 2014). Pyroptosis begins with activation of the NOD‐like receptor family pyrin 3 (NLRP3) inflammasome and caspases to cleave GSDMD, interleukin‐1β (IL‐1β) and interleukin‐18 (IL‐18) into their biologically active subunits. Gasdermin has 5 isoforms that share highly conserved domains (Broz et al., 2020), and GSDMD has been identified as a key effector molecule in pyroptosis. GSDMD is prototypically cleaved by caspase‐1 via a canonical pathway to release GSDMD‐N from the auto‐inhibitory GSDMD‐C subunit (Liu et al., 2019). Caspase 11 (mice) and caspase 4/5 (humans) also act on GSDMD via noncanonical mechanisms independent of the NLRP3 inflammasome (Man & Kanneganti, 2015; Shi et al., 2015) (Liu et al., 2019). GSDMD‐NT subunits oligomerize to insert pores into the phospholipid cell membrane to release cytokines (Evavold et al., 2018; Shi et al., 2015) and cause cell death (Liu et al., 2021), or the inner mitochondrial membrane to augment the release of reactive oxygen species (Wen et al., 2018) and initiate caspase‐3‐mediated apoptosis (Rogers et al., 2019). Previous studies have demonstrated increased GSDMD cleavage with ischemia reperfusion injury (IRI) and cisplatin AKI models (Linkermann et al., 2022; Miao et al., 2019) but surprisingly increased renal injury with a GSDMD knock‐out model (Linkermann et al., 2022). The single nucleotide polymorphism *I105N* in GSDMD has previously been characterized as a hypomorphic mutation (Aglietti et al., 2016), resulting in impaired GSDMD‐NT pore formation (Kayagaki et al., 2015). In these studies, we assessed AKI in mice harboring this GSDMD mutation and then assessed whether its role in determining renal injury rests with circulating cells or the kidney parenchyma. Finally, we assessed whether a pharmacological inhibitor of GSDMD function identified in drug repurposing studies protects against AKI.

## 2 METHODS

### 2.1 Animals

Protocols were approved by the Western Sydney Local Health District Animal Ethics Committee (protocol #4277). GSDMD mutant mice with the *I105N* mutation (Kayagaki et al., 2015) were supplied by the Australian Phenomics Facility (Australian National University). Studies were performed in accordance with the Australian code for the care and use of animals for scientific purposes developed by the National Health and Medical Research Council of Australia.

### 2.2 Ischemia reperfusion injury

Renal ischemia reperfusion injury (IRI) was performed as published previously (Rogers et al., 2012). Briefly, 10–12‐week‐old male mice were anesthetized using isoflurane/oxygen titrated to effect, with body temperature maintained at 36°C. A midline abdominal incision was performed and microaneurysm clamps were placed to occlude both renal pedicles for 20 min, after which time the clamps were removed. The abdomen was closed with 5/0 monofilament suture. In separate experiments, C57BL/6 mice were subjected to intraperitoneal injections of MCC950 (Sigma Aldrich, Burlington, MA, 10 mg/kg in 0.2 mL PBS) or disulfiram [Sigma Aldrich, 25 mg/kg or vehicle control (ethanol)] in 2 divided doses 12 h and 1 h prior to IRI. All mice were euthanized after 24 h reperfusion; blood was collected via cardiac puncture, kidney tissue was snap frozen, embedded and frozen in optimal cutting temperature (OCT) compound, or fixed in 10% neutral buffered formalin. For electron microscopy, systemic perfusion with 2.5% glutaraldehyde was performed postmortem.

### 2.3 Generation of chimeric mice

Bone marrow (BM) was harvested from donor mice as published previously (Rogers et al., 2012). Bone marrow (BM) was collected from male, age‐matched GSDMD^I105N/I105N^ homozygous mice or littermate controls by flushing femurs and tibiae with ice‐cold HBSS. Recipient mice were exposed to 10Gy whole body irradiation (X‐RAD320 machine, Precision X‐ray, CT), and freshly isolated BM (1×10e7 cells) was administered via retro‐orbital injection. Recipient mice were provided 0.2% neomycin water for the first 4 weeks, then challenged with bilateral IRI at 8 weeks.

### 2.4 Histological staining and injury scoring

Kidneys embedded in paraffin were sectioned at 4 μm and stained with hematoxylin and eosin by standard methods (Rogers et al., 2016). Sections were scored by two blinded, independent observers assessing five randomly selected corticomedullary areas (magnification ×100) per tissue section, as published previously (Rogers et al., 2012).

### 2.5 TUNEL staining

OCT‐embedded tissue was sectioned at 5 μm thickness and stained with TMR‐red TUNEL in situ cell death detection kit (Roche, Basel, Switzerland). Images were acquired using the Olympus FV1000 confocal laser scanning microscope (Olympus). TUNEL‐positive cells in five regions of interest per section were averaged.

### 2.6 Analysis of renal function and serum cytokine expression

Renal function was determined by measurement of serum creatinine using the Siemens Dimension Vista® System at the Institute of Clinical Pathology and Medical Research (Westmead Hospital). Serum was measured for pro‐inflammatory cytokines using the LegendPlex Mouse Inflammation Panel and analyzed using manufacturer software (BioLegend, San Diego).

### 2.7 qPCR analysis

RNA was extracted using the Isolate II RNA Mini Kit (Bioline, UK), quantified using a Nanodrop (BioTek, Winooski, VT), and reverse‐transcribed using a SensiFAST cDNA synthesis kit (Bioline). cDNA was amplified in triplicate using the CFX384 real‐time PCR machine (Bio‐Rad) with SensiFAST No‐ROX (Bioline) and targeted TaqMan primers (ThermoFisher, Waltham, MA). The following primers were used: hypoxanthine guanine phosphoribosyl transferase (HPRT1) Mm01324427_m1, tumor necrosis factor alpha (TNFα) Mm00443258_m1, interleukin 1beta (IL‐1β) Mm00434228_m1, interleukin 6 (IL‐6) Mm00446190_m1, Chemokine (C‐C motif) ligand 2 (CCL2) Mm00441242_m1, chemokine (C‐X‐C motif) ligand 2 (CXCL2) Mm00436450_m1, and chemokine (C‐C motif) ligand 5 (RANTES, CCL5) Mm01302428_m1. Thermal cycling conditions were 95°C for 2 min, followed by 40 cycles of 95°C for 15 s and 60°C for 1 min. Data were analyzed using the ΔΔCT method with expression normalized to the housekeeping gene (HPRT1), and littermate control (GSDMD^+/+^), GSDMD^+/+^ chimeric mice, or PBS‐treated animals were used as the reference controls.

### 2.8 Transmission electron microscopy

Kidney samples were washed with 0.1 M phosphate buffer, incubated in 2% osmium tetroxide (0.1 M cacodylate buffer), then in 2% uranyl acetate solution, and dehydrated through graded ethanol rinses before resin embedding. Resin blocks were cut and placed on copper grids for post‐staining with 2% uranyl acetate and lead citrate, with image acquisition using (JEM‐1400 Flash Electron Microscope, JOEL, Tokyo). Five regions of interest from post‐IRI kidneys were assessed for autophagic vesicles, as well as mitochondrial number and morphology using ImageJ (NIH, USA). Electron microscopy images were taken for mice bone marrow‐derived macrophages (BMDM), cultured in M‐CSF (Rogers et al., 2012) and stimulated with 100 ng/mL of lipopolysaccharide and 10 μM nigericin before fixation in 2.5% glutaraldehyde for 2 h.

### 2.9 Redox response

Superoxide (O_2_^•−^) was measured in particulate fractions from whole kidney using cytochrome c, by measuring the linear rate of superoxide dismutase (SOD, 150 U/mL, Sigma Aldrich) inhibitable cytochrome c reduction quantified at 550 nm using an extinction coefficient of 21.2 mM^−1^ cm^−1^ (Biotek Synergy 4 Hybrid Multi‐Mode Microplate Reader) (Meijles et al., 2017; Yao et al., 2014). Hydrogen peroxide (H_2_O_2_)‐generating activity was determined in whole kidney tissue using the Amplex Red (Invitrogen, CA) reaction monitored at 25°C (15 min); the emission increase was linear during this interval. To confirm the H_2_O_2_ signal, catalase (300 U/mL, Sigma Aldrich) was added in parallel wells, and the catalase‐inhibitable rate of H_2_O_2_ production was quantified from a H_2_O_2_ standard curve.

### 2.10 Statistical analysis

Data are represented as mean ± standard deviation unless otherwise stated, and analyzed with *t*‐test, Mann–Whitney *U* test, 1‐way or 2‐way ANOVA as appropriate, using Prism (v9, GraphPad). *p <* 0.05 was deemed significant.

## 3 RESULTS

### 3.1 GSDMD mutant mice were protected against AKI in a dose‐dependent manner

**FIGURE 1:**
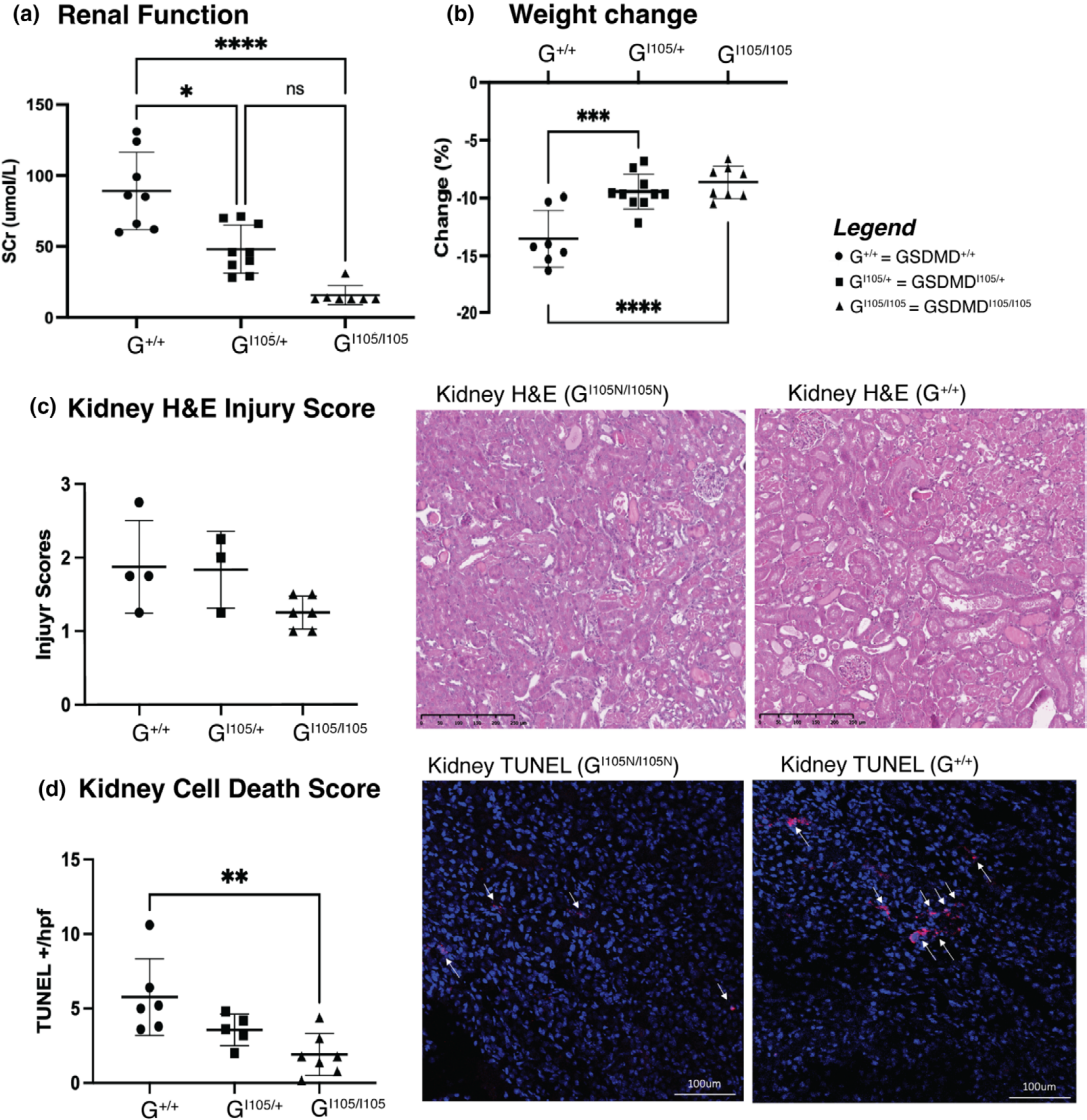
GSDMD^I105N^ variant mice show protection from AKI. Age‐matched, male littermate control (GSDMD^+/+^), heterozygous GSDMD^I105N/+^, and homozygous GSDMD^I105N/I105N^ mice were subjected to 20 min bilateral renal ischemia and 24 h reperfusion. (a) Serum creatinine and (b) change in weight of mice subjected to renal ischemia reperfusion injury (IRI). (c) Kidney tissue from mice subjected to renal IRI (*n* = 4–6 per group) was sectioned and stained with hematoxylin and eosin, followed by semiquantitative analysis of tissue injury from five corticomedullary regions of interest. Scale bars 250 μm. (d) TUNEL staining was performed on OCT‐embedded frozen sections of renal tissue and visualized using confocal microscopy. TUNEL^+^ (dead) cells/hpf were counted in five successive fields of +/+ or GSDMD^I105N/I105N^ mice. Images are shown as 200× original magnification, scale bars 100 μm. All data are mean ± SD. *p < 0.05, **p < 0.01, ***p < 0.001, ****p < 0.0001 by 1‐way ANOVA with Tukey's post hoc test.

**FIGURE 2:**
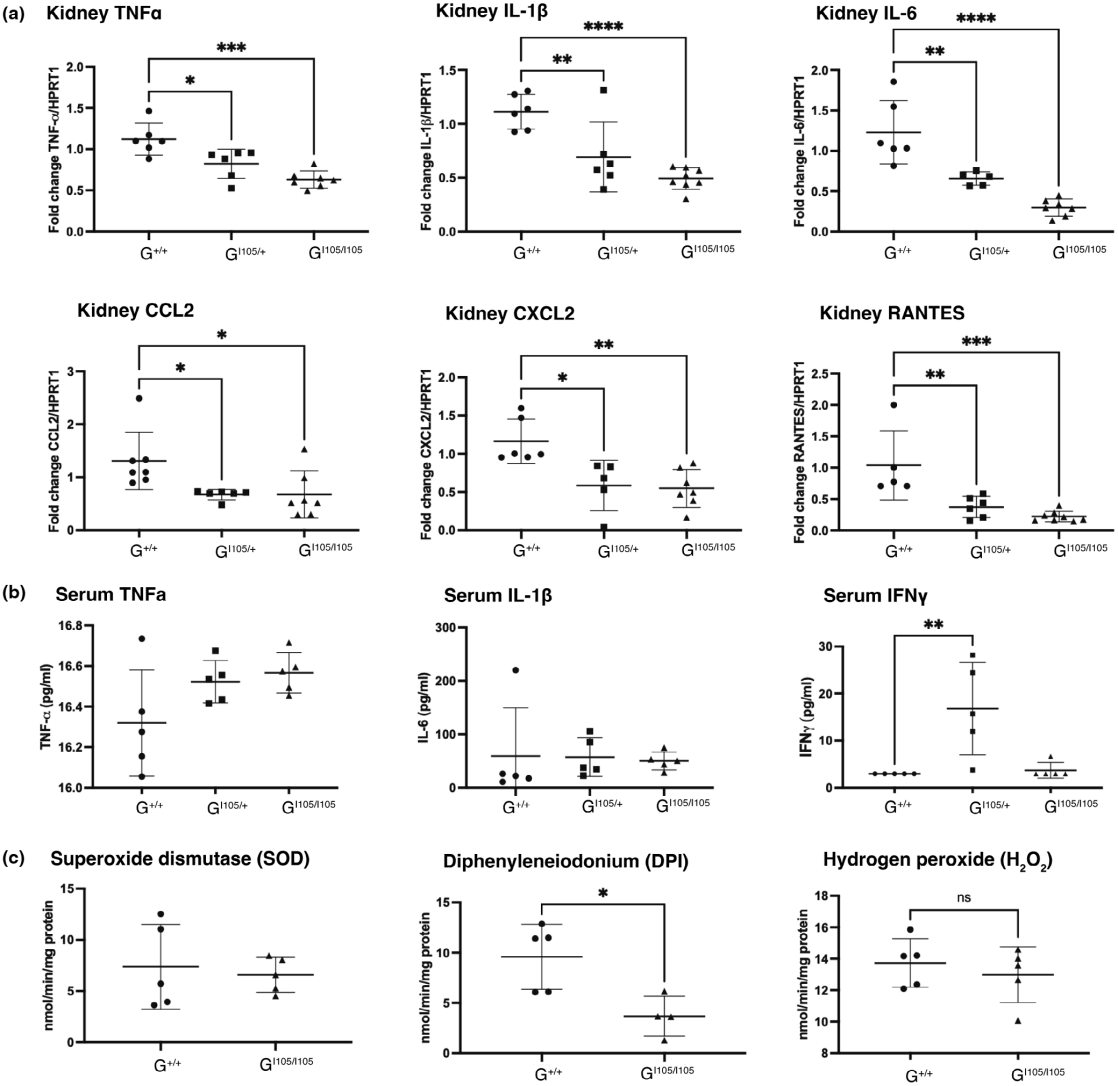
GSDMD^I105N^ variant mice protected from AKI demonstrate decreased parenchymal inflammatory signaling. Age‐matched, male littermate control (GSDMD^+/+^), heterozygous GSDMD^I105N/+^, and homozygous GSDMD^I105N/I105N^ mice were subjected to 20 min bilateral renal ischemia and 24 h reperfusion. (a) RNA isolated from kidney homogenates (*n* = 6–8 mice per group) was reverse transcribed and qPCR was performed for pro‐inflammatory factors TNF‐α, IL‐6, IL‐1β, CCL2, CXCL2, and RANTES. Results were normalized to the housekeeping gene HPRT1, and wild‐type (+/+) mice were used as the referent control. (b) Serum cytokine profile 24 h post ischemia reperfusion injury for TNF‐α, IL‐1β, and IL‐6. (c) Samples (*n* = 5 per group) were assessed for real‐time superoxide and hydrogen peroxide production using cytochrome c (following inhibition with diphenyleneiodonium, DPI) and amplex red assays, respectively. All data are mean ± SD; *p < 0.05, **p < 0.01, ***p < 0.001 by 1‐way ANOVA with Tukey's post hoc test.

GSDMD^I105N/I105N^ mice were protected from AKI compared to littermate controls, with significantly reduced serum creatinine (Figure 1a), reduced histological injury (Figure 1b) and less tubular cell death by TUNEL staining (Figure 1c). Heterozygous GSDMD^I105N/+^ mice exhibited a trend to an intermediate phenotype indicating a gene dosage effect. In keeping with the reduced inflammatory cell death, a broad reduction in pro‐inflammatory cytokine transcript expression in the kidney was demonstrated (Figure 2a), but with no difference in serum concentrations (Figure 2b). Analysis of oxidative stress showed decreased production of DPI‐related superoxide, and no change in hydrogen peroxide (Figure 2c) generation, suggesting a mitochondrial origin of the ROS moiety.

### 3.2 Transmission electron microscopy (TEM) reveals ultrastructural differences in GSDMD^I105N^ ^/I105N^ mice after AKI

**FIGURE 3:**
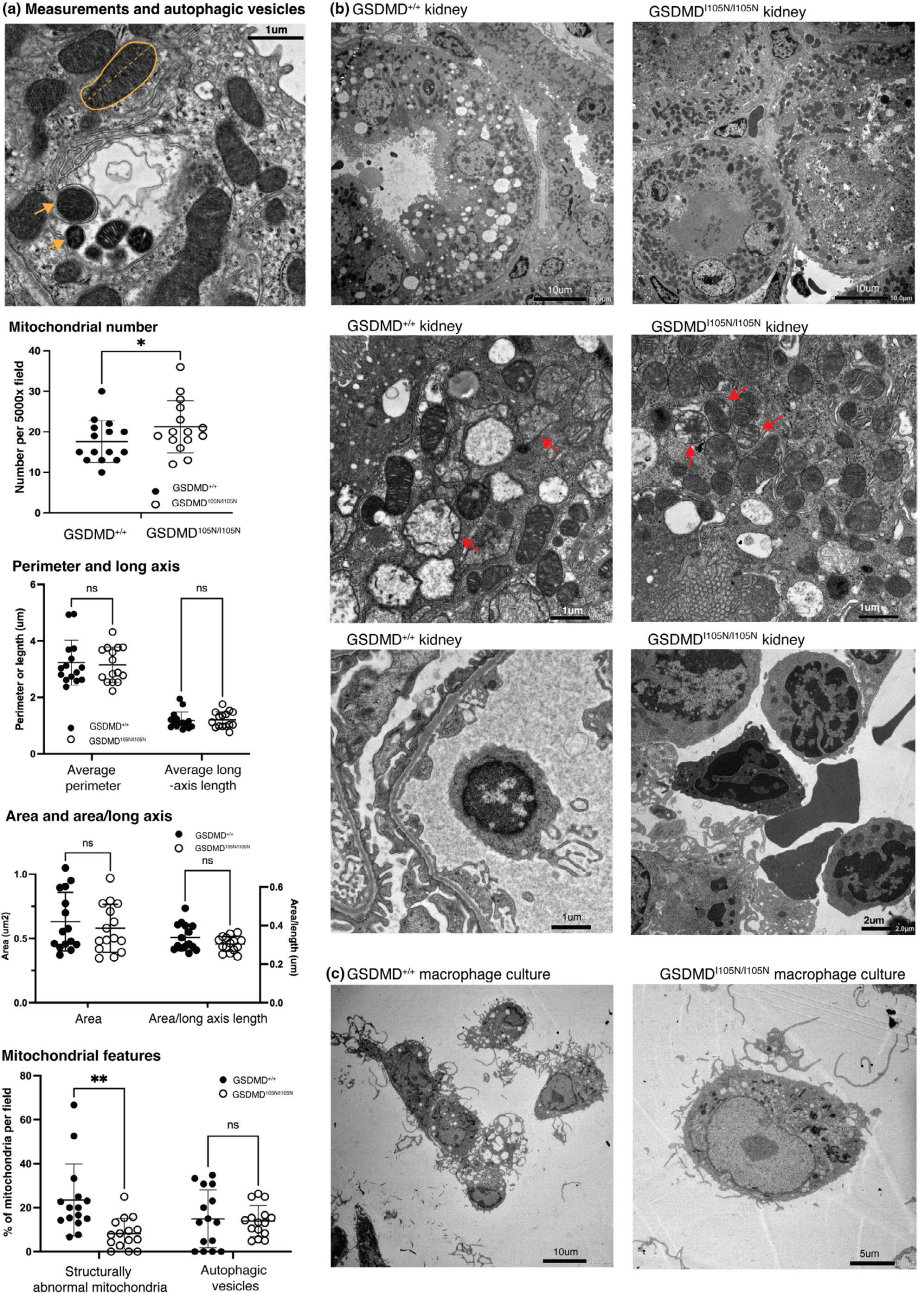
GSDMD^I105N^ variant mice show ultrastructural mitochondrial changes in response to AKI. (a) Whole kidney sections from littermate control (GSDMD^+/+^) mice analyzed by TEM demonstrate mitochondrial measurements for each high‐power field (5000× magnification, scale bar 1 μm). The *yellow outline* of the mitochondria was used to calculate the perimeter and the enclosed area; the *yellow dotted line* denotes the maximum long axis length of each mitochondrion, *yellow arrows* demonstrate autophagic vesicles, and r*ed arrows* are representative of structurally abnormal mitochondria. Comparison of mitochondrial numbers, mitochondrial perimeter, long axis length, mitochondrial area, ratio of mitochondrial area to long axis length, and number of autophagic vesicles between littermate control and homozygous mice. (b) Representative low and high magnification images of post‐IRI kidneys demonstrate a greater proportion of structurally abnormal mitochondria in the littermate controls (GSDMD^+/+^) compared to GSDMD^I105N/I105N^ mice (magnification 5000×, scale bar 1, 2, and 5 μm as shown). (c) Abundant membrane‐based pyroptotic bodies from GSDMD^+/+^ bone marrow‐derived macrophage (BMDM) following exogenous administration of lipopolysaccharide and nigericin, which were not replicated in BMDMs from GSDMD^I105N/I105N^ mice. All data are mean ± SD; *p < 0.05, **p < 0.01 by Student's *t*‐test (mitochondrial number) or 2‐way ANOVA with Tukey's post hoc test (perimeter, area, structural features).

TEM assessment of mitochondrial characteristics demonstrated fewer mitochondria per high power field and a greater proportion of mitochondria with structural abnormalities (swelling, abnormal cristae, and disrupted membranes) in littermate controls compared to GSDMD^I105N/I105N^ mice (Figure 3a). There was no significant difference in mitochondrial area, perimeter, long axis length, or ratio of area by long‐axis length, and autophagic vesicle numbers were similar between cohorts (Figure 3b). Pyroptotic bodies, previously described as pathognomonic EM findings (Chen et al., 2016), were not detected in RTEC and have not been described in non‐immune cells so far. BMDM showed clear differences in pyroptotic body formation, which was present in littermate controls but not GSDMD^I105N/I105N^ mice (Figure 3c).

### 3.3 Renal parenchymal gasdermin D determines pyroptosis and AKI risk

**FIGURE 4:**
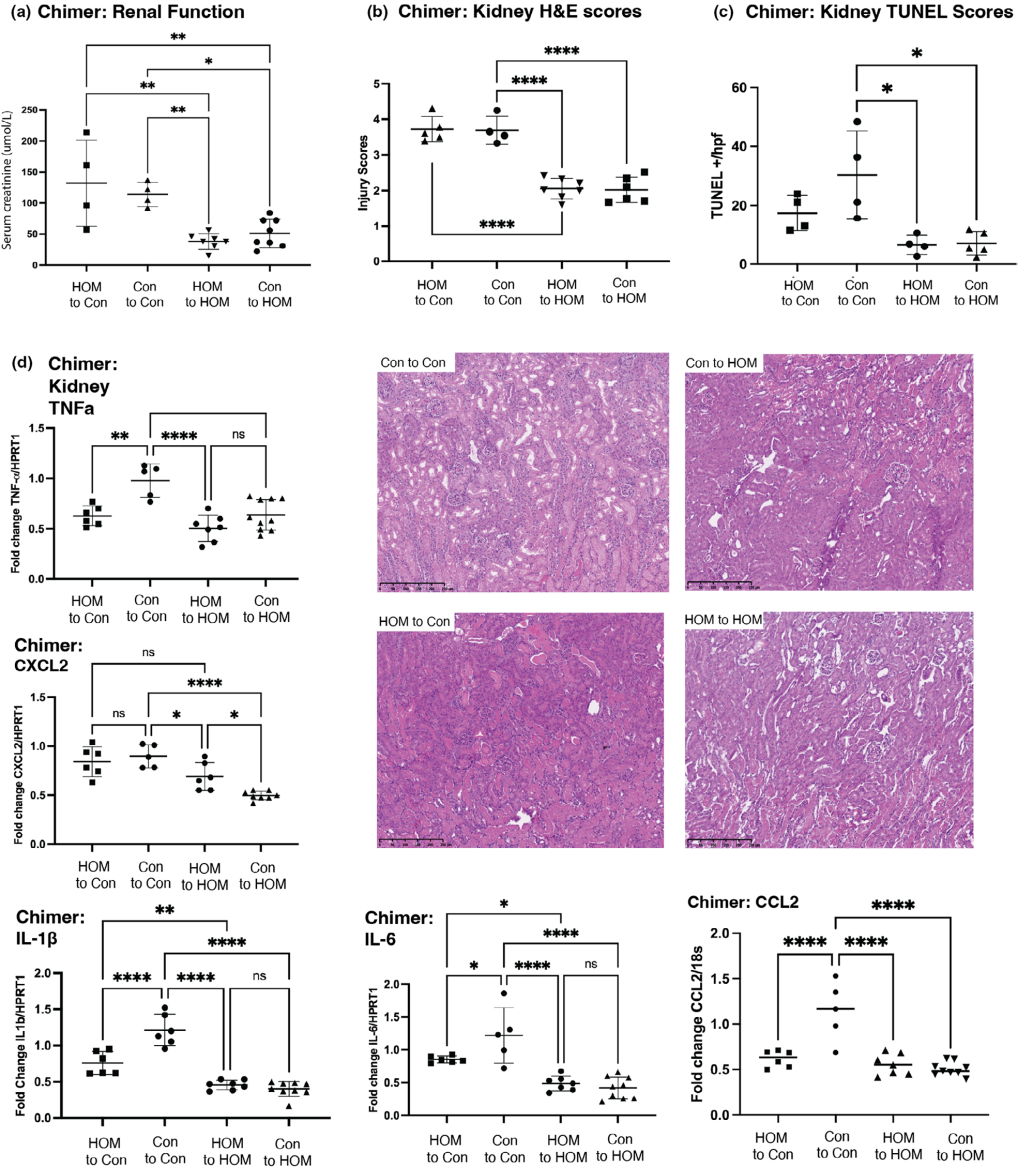
Manipulation of GSDMD signaling in renal parenchyma protects against AKI. Chimeric mice using GSDMD^+/+^ (Con) and homozygous GSDMD^I105N/I105N^ (HOM) mice were generated through syngeneic bone marrow transplantation and challenged with 20‐min bilateral renal ischemia and 24 h reperfusion. (a) Serum creatinine of mice subjected to renal ischemia reperfusion injury (IRI). (b) Kidney tissue from mice subjected to renal IRI (*n* = 6 per group) was sectioned and stained with hematoxylin and eosin, followed by semiquantitative analysis of tissue injury. Representative photomicrographs are shown (magnification 10×, scale bar ***). (c) TUNEL staining was performed on frozen sections of renal tissue and visualized using confocal microscopy. TUNEL^+^ (dead) cells/hpf were counted in five successive fields of +/+ or GSDMD^I105N/I105N^ mice. Representative photomicrographs are shown (magnification 10×, scale bar 250 μm). (d) RNA isolated from kidney homogenates (*n* = 5–8 mice per group) was reverse transcribed, and qPCR was performed for pro‐inflammatory factors TNF‐α, IL‐6, IL‐1β, CXCL2, and CCL2. Results were normalized to the housekeeping gene HPRT1, and GSDMD^+/+^ → GSDMD^+/+^ (Con to Con) mice were used as the referent control. Data shown are mean ± SD, *p < 0.05, **p < 0.01, ***p < 0.001, ****p < 0.0001 by 1‐way ANOVA with Tukey's post hoc test.

GSDMD is expressed on renal parenchyma (both epithelial and endothelial cells) which is the primary site of injury following IRI, as well as non‐parenchymal cells (macrophages, dendritic cells) that influx into the kidney in response to the release of danger‐associated molecular pattern molecules. To determine the relative roles of each compartment in the genesis of IRI, chimeric mice were generated and challenged with IRI. Recipient GSDMD^I105N/I105N^ mice had no significant difference in serum creatinine regardless of GSDMD^+/+^ or GSDMD^I105N/I105N^ BM donors (Figure 4a). Conversely, recipient GSDMD^+/+^ mice receiving either GSDMD^+/+^ or GSDMD^I105N/I105N^ BM had significantly higher serum creatinine compared to any GSDMD^I105N/I105N^ recipient chimera. Histological injury scores (Figure 4b) were similar between all groups, but TUNEL staining was significantly reduced in GSDMD^I105N/I105N^ recipient mice (Figure 4c). Pro‐inflammatory cytokine expression was reduced in GSDMD^I105N/I105N^ recipients compared to GSDMD^+/+^ recipients. Furthermore, the GSDMD^+/+^ recipient/GSDMD^I105N/I105N^ BM combination had lower TNF‐α, IL‐1β, and IL‐6 expression when compared to GSDMD^+/+^ recipient/GSDMD^+/+^ BM donors (Figure 4d) but this was insufficient to provide protection against IRI injury like GSDMD^I105N/I105N^ recipients.

### 3.4 Pharmacological inhibition of GSDMD, but not NLRP3, protects against AKI

**FIGURE 5:**
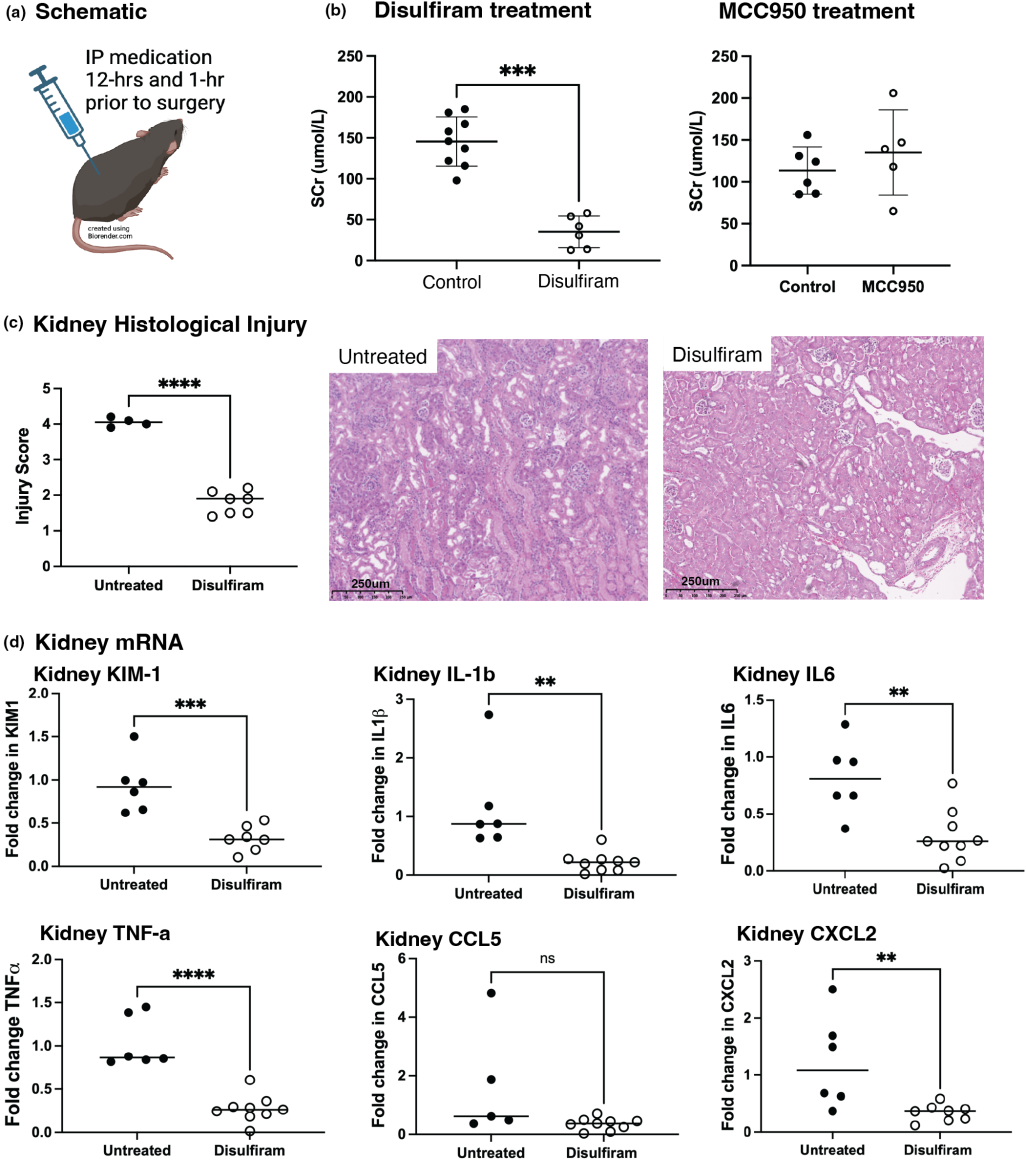
Pharmacological inhibition of GSDMD, but not the NLRP3 inflammasome, protects against AKI. (a) C57BL/6 mice were pre‐emptively treated with MCC950 (10 mg/kg). C57BL/6 mice were treated with disulfiram (25 mg/kg) prior to IRI surgery (created using BioRender.com). (b) Serum creatinine, (c) semiquantitative histological injury score based on H&E staining with representative photomicrographs (magnification 10×, scale bar 250 μm) and (d) analysis of pro‐inflammatory cytokine transcript expression. All data are mean ± SD; *p* < 0.05, **p < 0.01, ***p < 0.001, ****p < 0.0001 by Student's *t*‐test.

To test the effects of pharmacological inhibition of pyroptosis on AKI, we administered MCC950 (an inhibitor of NLRP3) or disulfiram (an inhibitor of GSDMD) (Hu et al., 2020) to C57BL/6 mice. Pre‐emptive dosing of MCC950 were not protected from IRI based on serum creatinine (Figure 5a). Disulfiram used at previously published doses (50 mg/kg) (Hu et al., 2020) was toxic to mice (with 50% pre‐AKI mortality), however, reduced dose of 25 mg/kg provided protection against severe AKI, with decreased histological injury (Figure 5b) and reduced pro‐inflammatory cytokine production (Figure 5c). Immunofluorescent staining demonstrated a reduction in LTL staining in vehicle‐treated AKI sections versus those treated with disulfiram. GSDMD staining was seen throughout the tubular and glomerular compartments (Figure 5d).

## 4 DISCUSSION

Regulation of cell death—particularly regulated necrosis of RTEC—is a primary facet of AKI. An exogenous insult that disrupts cell membrane integrity (autosis, ferroptosis, necroptosis, and pyroptosis) releases damage‐associated molecular patterns into the extracellular environment, stimulating a pro‐inflammatory innate immune response that balances cellular death in response to injury and subsequent repair. Our findings demonstrate that disruption of GSDMD function and the ability to execute pyroptosis can limit AKI severity following IRI. The ability of GSDMD to oligomerize and form pyroptotic pores in *I105N* mutant mice is impaired (Aglietti et al., 2016) and mice homozygous for this mutation demonstrated less renal injury—with lower serum creatinine, less RTEC death, and mitochondrial damage, as well as reduced pro‐inflammatory cytokine transcript expression.

Our results differ from the lack of ischemic renoprotection seen in some studies using GSDMD‐KO mice (Linkermann et al., 2022; Miao et al., 2019) and while this may reflect a difference in the functional effect of the point mutation versus genetic depletion, our results were supported by the renoprotective effect of pharmacological GSDMD inhibition using disulfiram. Both GSDMD and NLRP3 inflammasome machinery are expressed in murine (Miao et al., 2019) and human (Tonnus et al., 2022) RTEC. Our use of a GSDMD variant mouse produced hypomorphic—not reduced—GSDMD such that quantification of protein and cleaved fragments would not be a constructive readout.

Disulfiram is FDA‐approved as an alcohol‐sensitizing agent, but has been recently shown to limit pore‐forming activity associated with GSDMD cleavage (Hu et al., 2020). The dose used to successfully limit AKI was significantly reduced compared to the cited publication, resulting in animal mortality in our hands, although our time course between administration and exogenous insult was shorter and both doses are well below the cited LD_50_. Contrary to published literature (Franke et al., 2021; Zou et al., 2020), blockade of the NLRP3 inflammasome via MCC950 did not provide any protection against IRI, although our dosing was substantially less than these previous studies. Additional pharmacokinetic and pharmacodynamic studies are required to optimize dosing protocols.

Chimeric experiments demonstrated that limiting pyroptosis in renal parenchymal cells, rather than the immunological compartment, was critical to reducing cell death and injury following IRI. This is in keeping with the susceptibility to cell injury in highly metabolic active RTEC. The *I105N* mutation does not impair pro‐IL‐1β processing, but the role of the GSDMD‐N signaling in the non‐canonical inflammasome pathway driving caspase‐1‐dependent IL‐1β processing is unclear. Our results also showed a moderate reduction in whole kidney IL‐1β transcript, but not the IL‐1β serum concentration in keeping with its local effect. GSDMD pores can insert into the inner mitochondrial membrane to augment cell death, and this could be a contributing factor to reduced cell death. RTEC mitochondria from GSDMD^I105N/I105N^ mice displayed less damage and structural abnormalities compared to control mice.

Previous research supports a role for GSDMD in the development of septic shock following pathogenic Gram‐negative bacterial infection or exposure to LPS (Kayagaki et al., 2015) through caspase‐11‐mediated cleavage in macrophages. Studies interrogating the role of GSDMD in nonimmune cells in AKI have been met with discrepant results, with GSDMD‐deficient mice demonstrating both protection (Miao et al., 2019) and exacerbation (Tonnus et al., 2022) of injury. This difference may be due to the model of tubular insult employed, noting that the degree of IRI (based on serum BUN/creatinine) was mild (Tonnus et al., 2022) and cisplatin leads to cumulative RTEC injury over days (Miao et al., 2019; Tonnus et al., 2022) with negligible difference at 24 h post‐injury.

Numerous drugs and chemical compounds have been repurposed for a potential therapeutic benefit in IRI, and our study adds to the recent data demonstrating that inhibiting GSDMD is a useful approach. Understanding the mechanism(s) of pro‐inflammatory cell death adds to the complexity of AKI. However, despite all identified potential therapeutics to date, none have transformed or even reached routine clinical use despite compelling findings in pre‐clinical studies and early‐phase human trials investigating the treatment of AKI. This lack of success suggests the IRI community requires additional translational approaches to bridge the treatment gap.

## AUTHOR CONTRIBUTIONS

J.S.Y.L., S.I.A., and N.M.R. designed the research. J.S.Y.L. and N.M.R. performed the animal experiments. J.S.Y.L., A.M., D.N.M., K.T., S.M.J., K.S., and N.M.R. performed tissue analysis. J.S.Y.L., S.I.A., and N.M.R. wrote the manuscript. All authors approved the final version of the manuscript.

## FUNDING INFORMATION

JSYL is a recipient of the National Health and Medical Research Council (NHMRC) postgraduate scholarship (GNT116877) and the Westmead Association BJ Amos Travelling Scholarship. NMR is supported by a NHMRC Investigator Grant (GNT2007991).

## CONFLICT OF INTEREST STATEMENT

The authors declare no conflict of interest.

## ETHICS STATEMENT

The animal ethics protocol was approved by the Western Sydney Local Health District Animal Ethics Committee (protocol #4277).
