# Correction to “Gasdermin D mutation protects against renal ischemia reperfusion injury”

**DOI:** 10.14814/phy2.70819

**Published:** 2026-03-17

Li, J. S. Y., Moudgil, A., Meijles, D. N., Shaw, K., Julovi, S. M., Trinh, K., Alexander, S. I., & Rogers, N. M. (2025). Gasdermin D mutation protects against renal ischemia reperfusion injury. *Physiological Reports*, 13, e70254. https://doi.org/10.14814/phy2.70254.

The American Physiological Society and The Physiological Society (UK) are issuing a Society Note to include an Editor Disclosure Statement that was accidentally omitted from this published article in *Physiological Reports*. It should have been part of the Conflict of Interest statement.

Editor Disclosure Statement

Natasha M. Rogers, a co‐author on this article, was an Associate Editor of the journal at the time the article was published. The Societies verify that Natasha M. Rogers was not involved in the handling of the manuscript and did not have access to information regarding the peer‐review process or final disposition of the article. An alternate editor oversaw the peer‐review and decision‐making processes.

